# Gene Ontology and Expression Studies of Strigolactone Analogues on a Hepatocellular Carcinoma Cell Line

**DOI:** 10.1155/2019/1598182

**Published:** 2019-08-04

**Authors:** Mohammed Nihal Hasan, Syed Shoeb Razvi, Hani Choudhry, Mohammed A. Hassan, Said Salama Moselhy, Taha Abduallah Kumosani, Mazin A. Zamzami, Khalid Omer Abualnaja, Majed A. Halwani, Abdulrahman Labeed Al-Malki, Jiannis Ragoussis, Wei Wu, Christian Bronner, Tadao Asami, Mahmoud Alhosin

**Affiliations:** ^1^Department of Biochemistry, Faculty of Science, King Abdulaziz University, Jeddah, Saudi Arabia; ^2^Department of Biochemistry, All India Institutes of Medical Sciences, New Delhi, India; ^3^MS Research Foundation, Hyderabad, India; ^4^Department of Genetics, Vasavi Medical Research Center, Hyderabad, India; ^5^Cancer Metabolism and Epigenetic Unit, Faculty of Science, King Abdulaziz University, Jeddah, Saudi Arabia; ^6^Cancer and Mutagenesis Unit, King Fahd Medical Research Centre, King Abdulaziz University, Jeddah, Saudi Arabia; ^7^Department of Basic Medical Sciences, College of Medicine and Health Sciences, Hadhramout University, Mukalla, Yemen; ^8^Bioactive Natural Products Research Group, King Abdulaziz University, Jeddah, Saudi Arabia; ^9^Experimental Biochemistry Unit, King Fahd Medical Research Center, King Abdulaziz University, Jeddah, Saudi Arabia; ^10^Biochemistry Department, Faculty of Science, Ain Shams University, Cairo, Egypt; ^11^Production of Bioproducts for Industrial Applications Research Group, King Abdulaziz University, Jeddah, Saudi Arabia; ^12^Nanomedicine Department, King Abdullah International Medical Research Center (KAIMRC), King Saud bin Abdulaziz University for Health Sciences, Riyadh, Saudi Arabia; ^13^Department of Human Genetics, McGill University, Montréal, QC, Canada H3A 0C7; ^14^McGill University and Genome Québec Innovation Centre, Montréal, QC, Canada H3A 1A4; ^15^Department of Medicine, University of California, San Francisco, CA 94143, USA; ^16^Institut de Génétique et de Biologie Moléculaire et Cellulaire (IGBMC), INSERM U1258 CNRS UMR 7104, Université de Strasbourg, Illkirch, France; ^17^Graduate School of Agricultural and Life Sciences, The University of Tokyo, Bunkyo, Tokyo 112-8657, Japan

## Abstract

Human hepatocellular carcinoma (HCC) is the most common and recurrent type of primary adult liver cancer without any effective therapy. Plant-derived compounds acting as anticancer agents can induce apoptosis by targeting several signaling pathways. Strigolactone (SL) is a novel class of phytohormone, whose analogues have been reported to possess anticancer properties on a panel of human cancer cell lines through inducing cell cycle arrest, destabilizing microtubular integrity, reducing damaged in the DNA repair machinery, and inducing apoptosis. In our previous study, we reported that a novel SL analogue, TIT3, reduces HepG2 cell proliferation, inhibits cell migration, and induces apoptosis. To decipher the mechanisms of TIT3-induced anticancer activity in HepG2, we performed RNA sequencing and the differential expression of genes was analyzed using different tools. RNA-Seq data showed that the genes responsible for microtubule organization such as TUBB, BUB1B, TUBG2, TUBGCP6, TPX2, and MAP7 were significantly downregulated. Several epigenetic modulators such as UHRF1, HDAC7, and DNMT1 were also considerably downregulated, and this effect was associated with significant upregulation of various proapoptotic genes including CASP3, TNF-*α*, CASP7, and CDKN1A (p21). Likewise, damaged DNA repair genes such as RAD51, RAD52, and DDB2 were also significantly downregulated. This study indicates that TIT3-induced antiproliferative and proapoptotic activities on HCC cells could involve several signaling pathways. Our results suggest that TIT3 might be a promising drug to treat HCC.

## 1. Introduction

In 2012, 0.8 million patients were diagnosed with liver cancer, the seventh highest age-related incidence rate globally [1]. Human hepatocellular carcinoma (HCC) is the most common type of primary liver cancer in adults, as well as the most frequently recurrent malignancy without any effective therapy [2, 3]. HCC is the third most common cancer-related cause of mortality globally [1]. Several etiological factors could lead to the development of HCC including hepatitis B virus (HBV), hepatitis C virus (HCV), alcohol, cirrhosis, and nonalcoholic fatty liver disease (NAFLD) [1].

There exists an inveterate history of compounds, derived from plants, serving as anticancer agents [4]. These compounds can exert their inhibitory effects on cancer cells by targeting several pathways including cell cycle arrest, cell proliferation, and apoptosis. Strigolactones (SLs) are a novel class of phytohormones, which control the branching of shoot architecture by hindering growth and self-renewal of axillary meristem cells [5, 6]. It has previously been reported that synthetic SL analogues instigate G2/M cell cycle arrest and apoptosis by regulating the p38 and JNK1/2 MAPKs signaling pathways, causing the induction of stress in an array of solid and nonsolid human cancer cells, including prostate, colon, leukemia, osteosarcoma, and lung cancer cell lines.

It has only slight effects on the growth, survival, and viability of nontransformed human fibroblasts, healthy primary prostate cells, and mammary epithelial cells [7, 8]. SL analogues demonstrated their anticancer effects in a xenograft model of breast cancer [9] and have also been shown to affect the integrity of the microtubule network by impeding the migration of highly invasive breast cancer cell lines [9].

Recently, SL analogues have been found to destabilize the genomic DNA of cancer cells by inducing DNA double-strand breaks (DSBs) and activating the DNA damage and simultaneously hindering DNA repair, resulting in cell death. It is noteworthy that these activities of SL analogues have not been reported in nontransformed BJ fibroblast cells [10]. Furthermore, the efficiency of the delivery of SL analogues (hence their therapeutic efficacy) to prostate cancer cells may get enhanced by encapsulation of SL analogues in glutathione/pH-responsive nanosponges [11].

Synthetic SL analogues have been reported to downregulate RAD51 expression through ubiquitination in a proteasome-dependent way, hence, reducing the localization of RAD51 to DSB sites [10]. RAD51 is a crucial component of a prominent DNA repair pathway, the homology-directed repair (HDR) machinery. Overexpression of RAD51 causes an increase in DNA repair activity, which could result in resistance to DNA damage that is usually imposed by radiotherapy or chemotherapy [12, 13].

**Figure 1:**
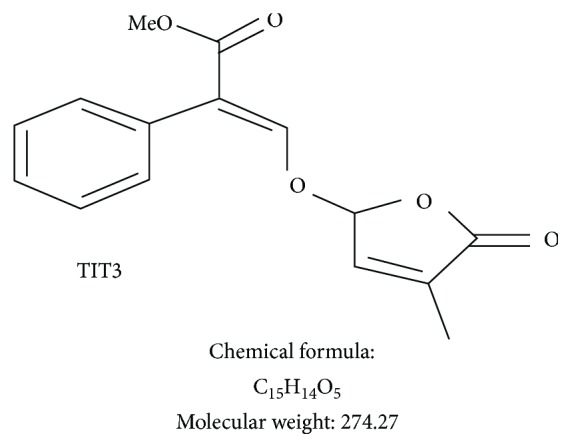
Molecular structure, chemical formula, and molecular weight of SL analogue TIT3.

In our previous study, we found that the newly synthesized SL analogue, TIT3, inhibits proliferation and induces apoptosis of HepG2 (hepatocellular carcinoma) cells with minimal effects on healthy cells [14]. The molecular structure of TIT3 is shown in Figure 1.

In the present study, we performed RNA sequencing and the differential expression of genes was analyzed using different tools to analyze and investigate the differential gene expression of HepG2 cells treated with TIT3 and to disclose the possible signaling pathways leading to the inhibition of cell proliferation and induction of apoptosis.

## 2. Materials and Methods

### 2.1. Cell Culture and Treatment

HepG2 cells were obtained from ATCC (Manassas, Virginia, USA). These cells were then sustained at 37°C, in a humidified incubator, at 5% CO_2_. DMEM (UFC Biotech, Riyadh, KSA) supplemented with 10% fetal bovine serum (FBS) (LifeTech, catalogue no: 16000-044) and 1% (100 U/ml) penicillin-streptomycin (LifeTech, catalogue no: 15140-122) was used to maintain cells.

### 2.2. RNA-Seq, Differentially Expressed Genes, and Bioinformatics Analysis

HepG2 cells were treated with 60 *μ*M of SL analogue TIT3 (IC_50_ of 63.46 *μ*M) [14] for 24 hours, in triplicate. An RNeasy kit (QIAGEN) was used to extract the total RNA, and the concentration of RNA was quantified. A bioanalyzer was used to analyze the quality of the total RNA (RIN score > 7.0). Sequencing libraries were then generated using TruSeq Stranded mRNA Sample Preparation Kits (Illumina, CA) from 2500 ng of the total RNA from each of the three replicates. The Illumina HiSeq 2000 system was used to conduct 50 bp long single-end deep sequencing. The FASTX-Toolkit was used to remove the adaptor sequence and filtering of low-quality base call and low-quality reads. The short filtered sequencing reads that were acquired were mapped to the human genome by the TopHat2 and Subreads package; the featureCounts function was used to quantify the gene expression values. These gene expression values were then used to calculate the size of the library and dataset dispersion for the analysis of differentially expressed genes [15]. Differential gene expression was examined using the R/Bioconductor package edgeR and established by log fold change (LogFC) and false discovery rate (FDR) (LogFC ≥ 1 or ≤-1; FDR ≤ 0.05).

### 2.3. Bioinformatics Analysis

The gene set functional analysis and pathway analysis were analyzed using the gene ontology (GO) and KEGG pathway. The gene IDs of interest were converted to EntrezID and uploaded to DAVID bioinformatics tools. GO and KEGG pathway analysis were performed by setting all the GO terms and KEGG pathway genes as background genes. Overrepresented GO terms or pathways are determined by the enrichment score (EASE ≤ 0.1, gene count ≥ 2).

## 3. Results

### 3.1. Gene Expression Is Regulated by TIT3

Data obtained from HepG2 cells treated with 60 *μ*M of SL analogue of TIT3 revealed that the mRNA expression of 3240 genes was modulated, with 1473 genes being upregulated (log fold change < 1.5; *p* < 0.05) and 1767 genes being downregulated (log fold change>−1.5; *p* < 0.05). The number of altered transcripts has been organized based on the log fold change (LogFC) or the *p* value (Tables 1 and 2). Overall, the number of transcripts being upregulated was fewer than the number of transcripts being downregulated.

### 3.2. Gene Enrichment Analysis of Altered Transcripts

**Figure 2:**
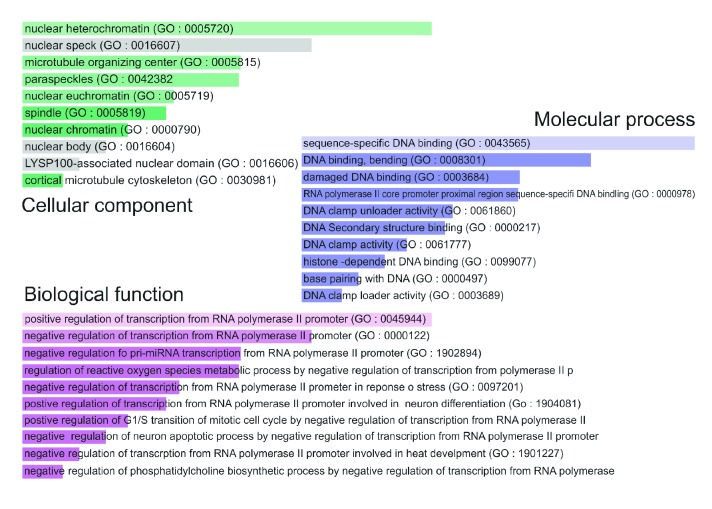
Gene ontology (GO) of upregulated genes in TIT3-treated HepG2 cells. The bar length represents the significance of that specific gene set or term, and the degree of the brightness of the color denotes the significance (*p* < 0.001) of the differentially expressed genes.

**Figure 3:**
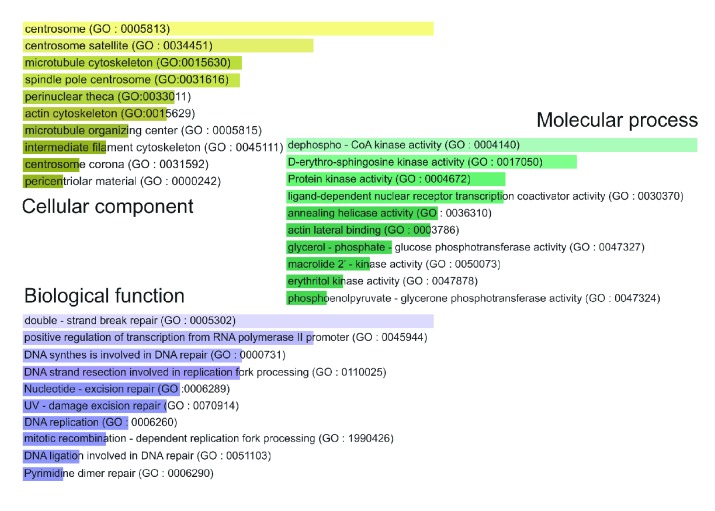
Gene ontology (GO) of downregulated genes in TIT3-treated HepG2 cells. The bar length represents the significance of that specific gene set or term, and the degree of the brightness of the color denotes the significance (*p* < 0.001) of the differentially expressed genes.

The gene enrichment analysis of gene ontology (GO) terms (*p* < 0.0001) revealed that there was a significant increase in the negative regulation of transcription by the RNA polymerase II promoter and negative regulation of G1/S transition of mitosis and a substantial decrease in the damaged DNA repair genes. A summary of GO analysis with different biological processes, cell components, and molecular functions of upregulated and downregulated transcripts in HepG2 cells treated with TIT3 is shown in Figures 2 and 3, respectively.

### 3.3. KEGG Pathway Analysis

**Figure 4:**
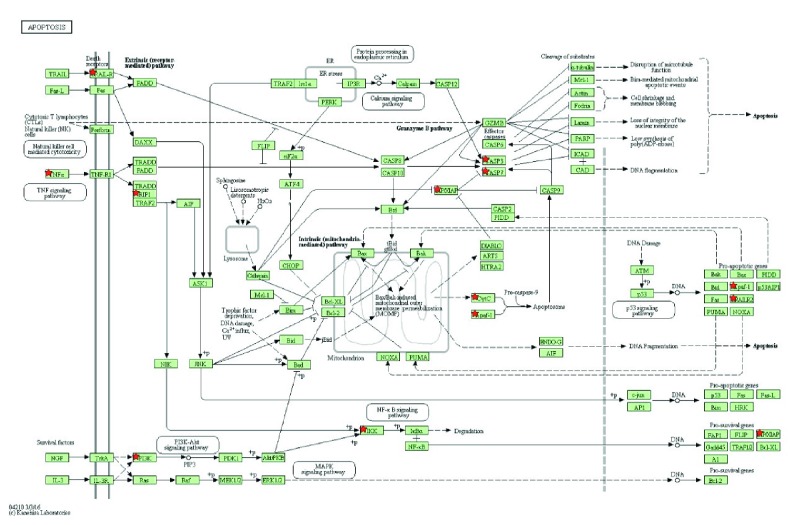
Analysis of the KEGG pathway in HepG2 cells after treatment with TIT3 illustrating the upregulated genes in apoptosis pathways; the genes regulated are marked.

**Figure 5:**
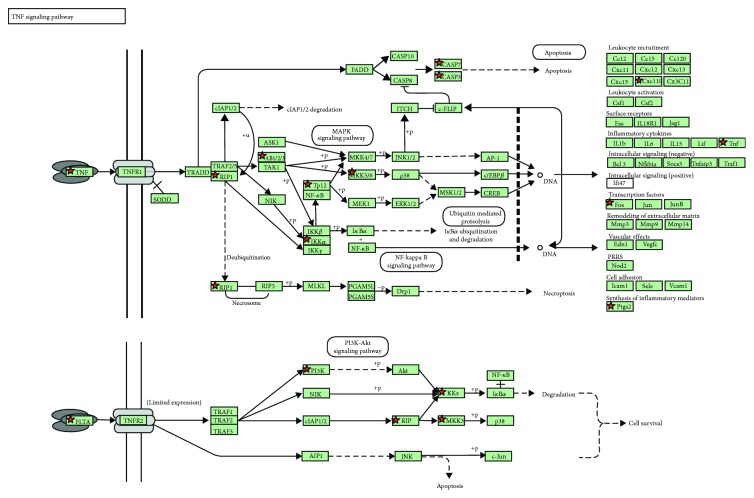
KEGG pathway analysis for HepG2 cells depicting the upregulated genes in the TNF signaling pathway after the treatment with TIT3; the altered genes are marked.

**Figure 6:**
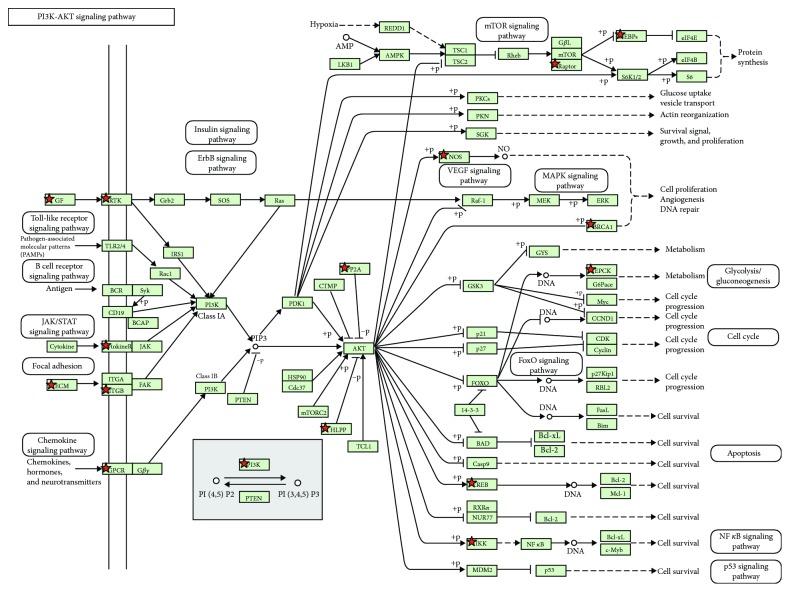
Significantly downregulated genes in the PI3K/Akt pathway after the treatment of HepG2 cells with TIT3 in this KEGG pathway analysis; the depicted downregulated genes are marked.

The KEGG pathway analysis revealed the probability of the involvement of apoptosis pathways involving TNF and PI3K-Akt (Figures 4–6). There was a significant decrease in the genes involved in the organization of microtubules such as BUB1B, TUBB, TUBG2, TUBGCP6, TPX2, and MAP7 (LogFC<−2.0; *p* < 0.0001). A significant decrease was also found in the expression levels of crucial epigenetic players UHRF1, DNMT1, and HDAC7, known to inhibit the expression of several tumor suppressor genes in cancer (LogFC<−1.7; *p* < 0.001).

**Figure 7:**
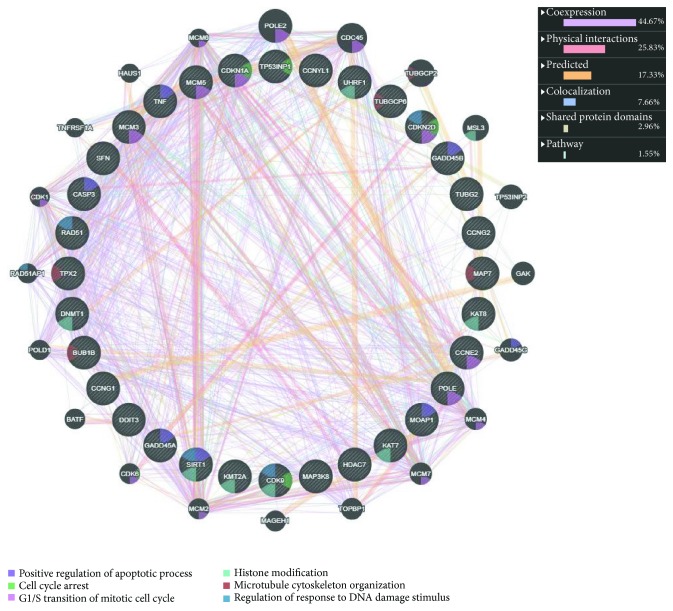
Outline of the interactions of different altered genes with their functions in HepG2 cells after treatment with TIT3.

**Figure 8:**
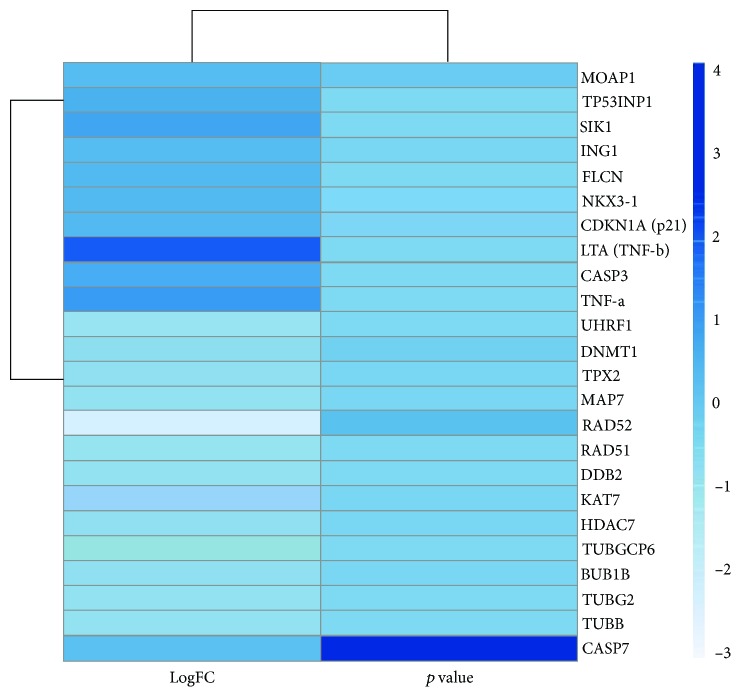
The heat map of the significantly deregulated genes represents the change with the intensity of the variation in color; with the alteration of LogFC (fold change) from -3 to +4 in TIT3-treated HepG2 cells as compared to untreated cells.

Additionally, genes responsible for DNA damage repair including DDB2, RAD51, and RAD52 were substantially downregulated (Table 3). The expression levels of several tumor suppressor genes such as NKX-3, FLCN, ING1, SIK1, and TP53INP1 (LogFC > 2.0; *p* < 0.001), and genes exhibiting proapoptotic activities such as TNF-*α*, LTA (TNF-*β*), CASP3, MOAP1, and CASP7 (LogFC > 1.6; *p* < 0.01) as well as genes having antiproliferative effects such as CDKN1A (p21) (LogFC > 2.2; *p* < 0.001) were significantly increased in response to TIT3 treatment (Table 4).  Figure 7 shows the probable interactions of genes of the different transcriptional regulators, and Figure 8 depicts a heat map, representing the comprehensive regulation of gene expressions in terms of their *p* values and LogFC.

## 4. Discussion

Synthetic SL analogues have been reported to induce cell cycle arrest and apoptosis in both solid and hematological tumors by targeting several signaling pathways [7, 8]. Our previous study showed that synthetic SL analogue TIT3 inhibited the proliferation and migration and induced apoptosis of HepG2 cells with minimal toxicity towards healthy noncancerous cells [14]. Since TIT3 impeded the migration of HepG2 cells, we suggested that such an effect is a result of the interference with the organization of the microtubular network. Data obtained from RNA-Seq showed significant downregulation of BUB1B, TUBB, TUBG2, TUBGCP6, TPX2, and MAP7 genes, which are known to be involved in the microtubular organization, suggesting that the antimetastatic and proapoptotic effects of TIT3 could be challenged by mechanisms involved in the organization of microtubules. Our results are consistent with several studies showing that SL analogues can induce apoptosis in breast cancer cells [9]. Other cancer cell lines such as melanoma, colon, lung, prostate, and osteosarcoma were also reported to be affected by SL analogues through targeting of the microtubular network [8].

Furthermore, our results showed that the histone deacetylase 7 (HDAC7) was downregulated in response to TIT3 treatment. Interestingly, the unusual activity of the histone deacetylases including HDAC7 has been reported in many types of cancers [16]. HDAC inhibitors such as TSA, SAHA, and MS-275 are useful in the chemotherapeutic regimens of many cancers including HCC and significantly inhibit cell proliferation, and migration/invasion induces cell cycle arrest and apoptosis of HCC [17]. Knockdown of HDAC7 led to G1/S arrest in different cancer cells through the upregulation of the cell cycle inhibitor CDKN1A (p21). Interestingly, an increase in the expression levels of p21 mRNA has also been observed in TIT3-treated HepG2 cancer cell lines [18]. All the evidences above suggest that TIT3 could act as a HDAC inhibitor causing cell cycle arrest through a p21-dependent mechanism inducing inhibition of HepG2 cell proliferation.

Moreover, UHRF1, a well-documented regulator of gene expression in cancer [19, 20], was downregulated by TIT3 suggesting that UHRF1 could be a potent target for TIT3 in HCC. In alignment with our results, UHRF1 overexpression has been demonstrated to cause tumorigenesis in different cancer types including HCC [21]. UHRF1 inhibition by using pharmacological compounds is associated with the reactivation of various tumor suppressor genes, thus suppressing the proliferation of cancer cells by inducing apoptosis [22].

Double-strand breaks (DSBs) are the most notable form of DNA damage, and once the DSBs are formed, cells may undergo either of the two repair mechanisms: nonhomologous end joining (NHEJ) or homology-directed repair (HDR) [23, 24]. Previous studies have revealed that cancer cells which lack HDR are quite sensitive to DNA-damaging agents [25]. Our data obtained from RNA-Seq showed that TIT3 induced the downregulation of damaged DNA repair genes including DDB2, RAD51, and RAD52. Therefore, TIT3 could be an inhibitor of DNA repair proteins. In support of our results, the evidence is available in the literature that SL analogues can hamper HDR and impair DSB repair [10].

Caspase 3 (CASP 3) is known as an executioner caspase in apoptosis resulting in the inhibition of proliferation of HepG2 cancer cells [26]. TNF-*α* is also known to modulate proliferation, differentiation, and apoptosis or necrotic cell death in several different cell types including HepG2 cancer cells [27, 28]. Our results illustrated that the primary genes responsible for apoptosis including CASP 3, CASP 7, TNF-*α*, TNF-*β*, and MOAP1 were significantly upregulated by TIT3 treatment on HepG2 cancer cells.

Overall, we propose that the inhibition of HepG2 cancer cell growth was due to an interplay of genes wherein the treatment of TIT3 significantly altered their expression levels. Altered gene expressions affected cell proliferation, cell cycle, metastasis, and apoptosis. TIT3 could also be an inhibitor of HDAC and can target the organization of the microtubular network as well as affect the genes involved in DNA repair.

## 5. Conclusion

We provided evidence that TIT3 targets several critical pathways in HepG2 cells. Therefore, to obtain and establish a deeper understanding of the molecular mechanisms exerted by TIT3, molecular biology techniques such as Western blotting, qPCR, microarray, and proteomics must be done to reveal the specific targets.

## Figures and Tables

**Table 1:** Classification based on *p* values; total number of transcripts altered in TIT3-treated HepG2 cells.

Range of *p* value of genes in the transcriptome	Number of upregulated transcript genes	Range of upregulated LogFC	Number of downregulated transcript genes	Range of downregulated LogFC
≤0.05	1026	+1 to +7	968	-6.63 to -1.5
≤0.01	293	+1.5 to 7.3	511	-7.28 to -1.5
≤0.001	154	+1.55 to 12.47	288	-7.99 to -1.5

**Table 2:** Classification based on log fold change (LogFC) values; total number of transcripts altered in TIT3-treated HepG2 cells.

LogFC of the genes in transcriptome	Number of transcript-upregulated genes	LogFC	Number of transcript-downregulated genes	Range of *p* values
+12.5 to +3	503	-8 to -3	290	≤0.05
+2.9 to +2	478	-2.9 to -2	693	≤0.05
+1.5 to +1.9	491	-1.5 to -1.99	810	≤0.05

**Table 3:** Downregulated genes in TIT3-treated HepG2 cells as compared with untreated cells.

Genes	LogFC	*p* value
BUB1B	-2.018	3.71*E*-05
TUBB	-2.247	3.03*E*-06
TUBG2	-2.271	8.41*E*-06
TUBGCP6	-2.582	6.82*E*-06
TPX2	-2.054	2.44*E*-05
MAP7	-2.185	4.42*E*-05
UHRF1	-2.627	4.63*E*-06
DNMT1	-1.867	1.16*E*-04
HDAC7	-2.103	4.96*E*-05
KAT7	-2.026	4.65*E*-05
DDB2	-2.351	5.80*E*-06
RAD51	-2.463	1.29*E*-05
RAD52	-7.507	3.78*E*-04

^∗^Fold change treated vs control.

**Table 4:** Upregulated genes in TIT3-treated HepG2 cells as compared with untreated cells.

Genes	LogFC	*p* value
CASP3	3.506	2.35*E*-11
CASP7	1.605	2.36*E*-03
TNF-*α*	4.686	6.96*E*-08
LTA (TNF-*β*)	8.148	1.02*E*-05
MOAP1	2.131	1.95*E*-04
CDKN1A (p21)	2.420	7.69*E*-07
NKX3-1	2.522	1.89*E*-06
FLCN	2.556	5.82*E*-07
ING1	2.094	3.43*E*-05
SIK1	3.947	1.70*E*-13
TP53INP1	3.046	3.08*E*-09

^∗^Fold change treated vs control.

## Data Availability

The data used to support the findings of this study are available from the corresponding author upon request.
